# Erratum to “Decline of olfactory function in Parkinson’s disease: A ten-year longitudinal study”

**DOI:** 10.1177/1877718X251333920

**Published:** 2025-04-02

Roos DS, Klein M, Doty RL and Berendse HW. Decline of olfactory function in Parkinson’s disease: A ten-year longitudinal study. *Journal of Parkinson’s Disease* 2024; 14(7). DOI:10.1177/1877718X241298708.

Due to an inadvertent publication error, this article was mistakenly included in the print version of Volume 15, Issue 1, despite having already been published in Volume 14, Issue 7 in both print and online formats. To prevent duplicate online publication, it has been removed from the online version of Volume 15, Issue 1. The correct and citable version of this article remains as listed above.

